# Physical Forces Modulate Oxidative Status and Stress Defense Meditated Metabolic Adaptation of Yeast Colonies: Spaceflight and Microgravity Simulations

**DOI:** 10.1007/s12217-017-9588-z

**Published:** 2017-12-29

**Authors:** Timothy G. Hammond, Patricia L. Allen, Margaret A. Gunter, Jennifer Chiang, Guri Giaever, Corey Nislow, Holly H. Birdsall

**Affiliations:** 1 Durham VA Medical Center, Medicine Service Line, 508 Fulton Street, Durham, NC 27705 USA; 2 Nephrology Division, Department of Medicine, Duke University School of Medicine, Durham, NC 27710 USA; 3 Space Policy Institute, Elliott School of International Affairs, George Washington University, Washington, DC 20052 USA; 4 The Institute for Medical Research, Durham, NC 27705 USA; 5 Department of Pharmaceutical Sciences, The University of British Columbia, Vancouver, BC V6T 1Z3 Canada; 6 Department of Veterans Affairs, Veterans Healthcare Administration, Office of Research, Washington, DC 20420 USA; 7 Departments of Otorhinolaryngology, Immunology, and Psychiatry, Baylor College of Medicine, Houston, TX 77030 USA

**Keywords:** Yeast, Microgravity, Reactive oxygen species, Spaceflight, Shear stress, Random positioning machine, Apoptosis

## Abstract

Baker’s yeast (*Saccharomyces cerevisiae*) has broad genetic homology to human cells. Although typically grown as 1-2mm diameter colonies under certain conditions yeast can form very large (10 + mm in diameter) or ‘giant’ colonies on agar. Giant yeast colonies have been used to study diverse biomedical processes such as cell survival, aging, and the response to cancer pharmacogenomics. Such colonies evolve dynamically into complex stratified structures that respond differentially to environmental cues. Ammonia production, gravity driven ammonia convection, and shear defense responses are key differentiation signals for cell death and reactive oxygen system pathways in these colonies. The response to these signals can be modulated by experimental interventions such as agar composition, gene deletion and application of pharmaceuticals. In this study we used physical factors including colony rotation and microgravity to modify ammonia convection and shear stress as environmental cues and observed differences in the responses of both ammonia dependent and stress response dependent pathways We found that the effects of random positioning are distinct from rotation. Furthermore, both true and simulated microgravity exacerbated both cellular redox responses and apoptosis. These changes were largely shear-response dependent but each model had a unique response signature as measured by shear stress genes and the promoter set which regulates them These physical techniques permitted a graded manipulation of both convection and ammonia signaling and are primed to substantially contribute to our understanding of the mechanisms of drug action, cell aging, and colony differentiation.

## Introduction

The yeast *Saccharomyces cerevisiae* has broad genomic homology to human cells and is simple to grow and genetically manipulate (Lee et al. 2014; Kachroo et al. 2015; Nislow et al. 2015). This has made it a powerful model organism with biomedical applications to understand mechanisms of cell-to-cell interactions, cell survival, and aging (Cap et al. 2009a; Ayer et al. 2014; Herker et al. 2004). To survive, yeast in liquid cultures must use strategies that are very different from that used by yeast in agar-based colonies (Cap et al. 2009a). Survival in liquid culture is dependent on stress defense whereas survival in a colony is dependent on metabolic adaptation and stratification of the population in response to ammonia-mediated signaling (Cap et al. 2009a). Both modes of culture have applications for biomedical research. Chemical genomic assays using a deletion series of *Saccharomyces cerevisiae* grown in rich liquid media have provided powerful methods to identify the mechanism of action of known drugs and novel small molecules *in vivo* including chemotherapy anti-cancer agents (Smith et al. 2010). On the other hand, stratification and divergent metabolic adaption of yeast within a colony on solid agar models many of the changes seen within solid tumors (Cap et al. 2009a, 2010, 2012a, d).

Yeast is also a popular model for studies of cellular responses to microgravity and microgravity simulations. Multiple flight experiments have verified that *S. cerevisiae* remains fully viable, and responds to the microgravity environment with changes in metabolism [e.g. increase in phosphate uptake (Berry and Volz 1979) and phenotype [e.g. increase in number and distribution of bud scars (Walther et al. 1996)]. Studies of yeast in space have typically been conducted with liquid cultures. The responses of liquid culture yeast to real (e.g. spaceflight) microgravity and simulated microgravity include changes in reactive oxygen species, apoptosis, as well as shear defense mechanisms (Coleman et al. 2007, 2008a, b; Hammond et al. 1999; Johanson et al. 2002; Nislow et al. 2015). We have used a deletion series strategy, similar to chemical genomic assays, to demonstrate that mitochondrial and ribosomal redox gene pathways play a predominant role in the responses of yeast colonies to microgravity (Nislow et al. 2015). This genetic modulation of yeast colonies in real and simulated microgravity is dependent, at least in part, on shear stress promoters, apoptosis, and reactive oxygen species (Coleman et al. 2008a).

Little is known about the effects of spaceflight on yeast growing as a colony on agar. On Earth, yeast cells form giant multicellular colonies with characteristic organized morphologies (Cap et al. 2009b, 2012b, c). Around day nine, cells at the base of the colony, begin to apoptose and also begin to secrete ammonia. Ammonia signaling induces cells at the top and leading edges of the colony to reprogram their metabolic pathways and divide rapidly, thereby allowing the colony to continue to expand. Yeast colony stratification is indelibly linked to gravity-driven convection, as ammonia production and the gravity driven convection of produced ammonia mediate giant yeast colony differentiation (Cap et al. 2009a; Palkova et al. 1997). It is not known how the absence of convection seen in microgravity might affect this process.

To answer this question, we studied giant yeast colonies formed in true microgravity (e.g. spaceflight) and in two ground-based simulations of microgravity: rotation and random positioning. Rotating culture devices have been a popular model to mimic some of the physical factors induced by microgravity during spaceflight (Hammond and Hammond 2001; Lee et al. 2014; Birdsall et al. 2016). Redirecting gravity driven convection, by growing yeast on a vertical rather than horizontal agar slab, disrupts ammonia signaling, altering the redox status of yeast cells and gene expression kinetics during growth of giant yeast colonies (Birdsall et al. 2016). The random positioning machine (RPM), referred to as the 3-D clinostat, is a microgravity simulator based on the principle of ‘gravity-vector-averaging’, which can generate simulated gravity levels from 0g to 1g (Grimm et al. 2014). There is limited data comparing the RPM, simpler 2-D rotation, and true microgravity (Grimm et al. 2014), and controversy as to whether the forces they induce are distinct (Klaus et al. 2004; Murdoch et al. 2013; Wuest et al. 2017).

Metabolic adaption in giant yeast colonies is triggered by the depletion of nutrients in the agar and to enhance this process colonies are typically grown on nutrient-poor agar (Cap et al. 2009a; Palkova et al. 1997). In contrast, studies of yeast in liquid cultures, including chemical genomic assays, tend to use rich media. To evaluate the role of nutrients, we conducted parallel studies of yeast on both nutrient-rich (YPD) and nutrient poor (YE) media.

Three major analyzes were conducted in yeast grown as giant colonies under true and simulated microgravity. First, we measured the expression of a series of genes likely to be affected by microgravity using clones engineered to express enhanced green fluorescent protein (eGFP). Table 1 lists the clones used and the rationale for their selection. ATO3, INO1, SSA4, and RPL34b are known to be upregulated in different strata as yeast differentiate into giant colonies in ground studies (Cap et al. 2012a, d). BEM1 was included as an index of bud scarring, which is known to be altered in liquid cultures of yeast in spaceflight (Walther et al. 1996). TRR1 was included because giant colony differentiation on the ground and in rotation is accompanied by changes in redox (Birdsall et al. 2016; Cap et al. 2012d) and TRR1 is the key control enzyme in production of glutathione, the most abundant thiol buffering cellular reactive oxygen species (Trotter and Grant 2005). EDC3 was included as it is often constitutively expressed, proving a control for a field change effect (Kshirsagar and Parker 2004).

**Table 1:** Clones selected for study

Standard name	Systematic name	Description	Reason for selection
ATO3	YDR384C	Putative ammonium transporter	Upregulated in the upper cells of giant yeast colonies
INO1	YJL153C	Inositol-3-phosphate synthase	Upregulated in the lower cells of giant yeast colonies
TRR1	YDR353W	ThioRedoxin Reductase	Key control enzyme in redox which is altered in spaceflight
BEM1	YBR200W	Involved in budding	Bud scarring is altered in spaceflight in liquid cultures
EDC3	YEL015W	Enhancer of mRNA DeCapping	Constitutive control
RPL34B	YIL052C	Stress response gene	Upregulated in the lower cells of giant yeast colonies; Dependence on Msn4 and Sfp1 is altered in microgravity in liquid cultures
SSA4	YER103W	Stress response gene	Upregulated in the lower cells of giant yeast colonies; dependence on Msn4 and Sfp1 is altered in microgravity in liquid cultures
Sfp1	YLR403W	Stress-responsive transcriptional regulator	Differential effect on SSA4 and RPL34B in microgravity vs ground-based liquid cultures
Msn4	YKL062W	Stress-responsive transcriptional regulator	Differential effect on SSA4 and RPL34B in microgravity ground-based liquid cultures

Second, we evaluated the dependence of two stress response genes, SSA4 and RPL34b, on the stress promoters, Msn4 and Sfp1 (Johanson et al. 2002). We have previously shown that in liquid yeast cultures, SSA4 and RPL34b have novel transcription dependence on Msn4 and Sfp1 during spaceflight that differs from ground-based controls (Coleman et al. 2008a).

Third, we evaluated the viability and induction of apoptosis in the giant yeast colonies and the dependence of these outcomes on the stress promoters Msn4 and Sfp1. The differentiation of giant yeast colonies in terrestrial analyzes is dependent on the promoters Msn4 and Sfp1 (Cap et al. 2012b) but the effects of microgravity on this process are not known.

We have previously reported on the effects of media and rotation on the viability and redox potential in giant yeast colonies in ground-based studies (Birdsall et al. 2016). In this report, we extend analysis to include giant yeast colonies in the true microgravity of spaceflight as well as in the microgravity simulation by random positioning (RPM) and 2D rotation (Rotating Wall-Vessel head) positioning. Defining the utility of microgravity and microgravity simulations is confounded by use of different microgravity simulations, different media, and different outcomes. This study is a systematic examination of the effects of true microgravity versus common microgravity simulations, and nutrient rich media versus nutrient poor media, on major mechanistic mediators including shear stress promoters Msn4 and Sfp1, reactive oxygen species, and apoptosis. This lays the ground work for future yeast experiments to study cell-to-cell interactions, cell signaling, cell survival, colony differentiation and aging, mechanisms of drug action, and characteristics of tumor generation.

## Methods and Materials

### Chemicals and Reagents

*Saccharomyces cerevisiae* strain BY4743 (i.e., wild type (WT)), was used to match our earlier studies (Hammond et al. 2015). This parental strain, as well as Msn4 deletion, Sfp1 deletion, BEM1-eGFP and TRR1-eGFP yeast clones were purchased from Life Technologies (Grand Island, NY). FITC-VAD-fmk was purchased from Promega Corporation (Madison, WI). FITC Annexin V was purchased from Clontech Laboratories Inc. (Mountain View, CA). All other chemicals and reagents were purchased from Sigma-Aldrich (St. Louis, MO). Media consisted of “rich” YPD (1% yeast extract, 2% peptone, 2% dextrose (D-glucose)) or “poor” YE (1% yeast extract, 3% glycerol, 1% ethanol, and 10 mM CaCl_2_) (Birdsall et al. 2016). 2% agar was added to YPD or YE for solid phase cultures.

#### Construction of Yeast Green Fluorescent Protein (eGFP) Expression Clones

Synthetic oligonucleotides were used to direct integrate genomic of DNA coding for and in-frame GFP epitope tag at the C-terminus of selected open reading frames (ORFs) in the yeast genome as described previously (Howson et al. 2005) and https://yeastgfp.yeastgenome.org/.

#### Giant Yeast Colony Cultures: Static, Rotating Random Positioning Machine and Spaceflight

100 *μ* l of stationary culture inocula of each yeast strain were placed in 5 ml of fresh YPD or YE media and grown overnight in a shaking incubator at 30^∘^C. Omniwell plates prefilled with 40ml of YE or YPD agar were spotted with 60 *μ* l of the overnight liquid culture with forty-eight spots per plate. To minimize dehydration after drying the Omniwell lids were sealed to the base with Durapore tape and the plates were placed in plastic Ziploc™ bags. The plates were cooled to 4^∘^C and were divided into five sets of four plates - one set for each of the five growth conditions. Each set contained a full series on YPD agar and a full series on YE agar.

**Fig. 1:**
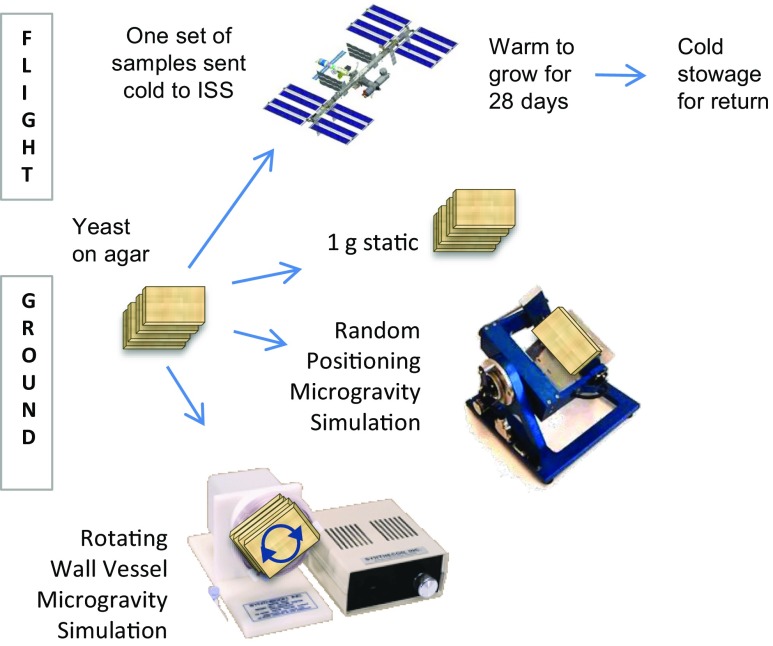
Four conditions were studied: Spaceflight and three forms of ground controls: static, rotating, and randomly positioned. Approximately two days post-launch, the plates on the ISS were warmed to 30 ^∘^C to initiate yeast growth. After 28 days inflight, the plates were chilled and returned to Earth. Static ground controls were oriented with the agar surface facing upwards. For rotation studies, plates were securely taped to the front of the rotating wall vessel head so that the plates were perpendicular to the floor. For random positioning, plates were secured to the platform of a Desktop Random Positioning Machine

Figure 1 summarizes the matrix of experiments performed. Flight and static ground control plates were sent to Kennedy Space Center in chilled shipping containers. Just prior to flight, working in a cold room, the plates were transferred to barcoded Ziploc™ bag and stacks of eight were placed in Lexan bags and then into plate habitats (PHABs; http://www-bioserve.colorado.edu/tech_Sheets_pdfs/SelectBioServeHardware.pdf; BioServe Space Technologies, CO). The PHAB is a sealed container that allows for gas exchange but meets NASA safety requirements for levels of containment. (https://www.nasa.gov/centers/johnson/engineering/life_support_systems/crew_payload/index.html). PHABs were placed in cold bags at 4^∘^C, and remained chilled for five days during handoff to the SpaceX-8 rocket. Flight samples were flown to the International Space Station (ISS). Approximately two days post-launch, the growth of the yeast on ISS was initiated by transferring the PHABs to Commercial Generic Bioprocessing Apparatus 4 (CGBA4) (Hoehn et al. 2004) that was set to 30 ^∘^C (BioServe Space Technologies, CO). After 28 days in flight, CGBA4 was commanded down to + 4 ^∘^C. The following day the PHABs were moved back to cold bags, and returned to Earth the next day on the Dragon capsule. Plates remained chilled for a total of six days during undocking, return to Earth, salvage, and transport to the lab for flow cytometry analysis.

The second set of plates, the static flight controls were loaded into a second set of PHABs at Kennedy Space Center and handled to match the exact timing and temperature profile of the flight samples. Ground controls were conducted with a 24-hour offset relative to the flight samples and the static controls in PHABs followed the same temperature changes as the flown samples.

A third and fourth set of samples were subjected to Wall-Vessel rotation or random positioning (RPM) during growth. The timing of the rotating and RPM cultures matched that of the flight samples with the exception that they were grown at ambient laboratory conditions (25 ^∘^C) rather than 30 ^∘^C and they were not placed in PHABs. To control for the difference, an additional set of static ground controls were maintained in plastic bags at 25^∘^C and also not placed in PHABs. Results from the two set of static controls, PHABs/30 ^∘^C versus no-PHAB/25 ^∘^C, did not differ (Pearson correlation > 95%) so data from the two were pooled in the statistical analyzes. Similarly, we could not detect any differences due to plate position of the replication using any of the modalities studied (Pearson correlation > 95%).

Static controls were incubated with the agar facing upwards. For rotation studies, four stacked Omniwell plates in plastic bags were securely taped to the front of the rotator head (Synthecon, Houston TX) oriented with the surface of the agar perpendicular to the floor (see Fig. 1). Plates were spun at 12 rpm in a direction co-planar with the surface of the agar around an axis of rotation at the center plate. Replicate colonies of each clone were spotted in 4 to 6 sites on the agar and located 10 to 75 mm from the axis of rotation. For random positioning, four Omniwell plates were taped to the baseplate of a Desktop Random Positioning Machine (Dutch Space, Leiden, The Netherlands), set to simulated 0 g during 3-D vector-averaging random positioning. The colonies on the agar surfaces were approximately 70mm, 55 mm, 40 mm, and 25 mm from the rotation axis. The stack was continuously re-positioned with random direction and speed to achieve a net zero gravity vector. Maximum acceleration/deceleration was 161 degrees/sec^2^, maximum input frame speed was 45 degrees/sec, and positioning speed was 45 degrees/sec. This data meets the Bonn Criteria (Hammond and Allen 2011).

Flight, rotation, and RPM growth conditions had four replicates on the parental strains and six replicates for the deletion strains. Static cultures had eight replicates for the flight, rotation, and RPM conditions and 12 replicates for the static cultures.

#### Assays for shear stress gene expression, cell death, gene expression, and reactive oxygen species

All assays were performed by flow cytometry. Our probes for viability, GFP expression, and yeast redox levels have been validated previously (Birdsall et al. 2016). The choice of parameters to assay were based on the importance of the effects of shear stress promoters, reactive oxygen species, and cell death in giant yeast colonies. On the day of each assay, giant yeast colonies were removed from the agar plate with a plastic tissue culture loop, placed in 2 ml of phosphate buffered saline (PBS), and suspended by vortex. 50 *μ* l aliquots of yeast in PBS were placed in 5ml tubes preloaded with 300 *μ* l of propidium iodide (PI) at 1 *μ* g/ml solution to measure viability, or 300 *μ* l of dihydroethidium (DHE) at 5 *μ* g/ml to measure reactive oxygen.

To measure caspase yeast were washed once in PBS, pelleted, incubated for 20 min at 28 ^∘^C in 200 *μ* l of 10 *μ* M FITC-VAD-fmk, and then washed once in PBS. To measure annexin, yeast were washed in sorbitol buffer (1.2M sorbitol, 0.5 mM MgCl2, 35 mM potassium phosphate, pH 6.8), digested for 30 min at 28 ^∘^C with 5.5% glusuase and 15 Um/ml lyticase in sorbitol buffer, and washed twice in kit binding buffer to which 1.2 M sorbitol is added. Yeast were resuspended in 100 *μ* l binding buffer with sorbitol and stained with 5 *μ* l of annexin and 1 *μ* l of PI at 0.1 mg/ml in binding buffer with sorbitol for 15 min at room temperature for 15 min. 400 *μ* l binding buffer with sorbitol was added and the samples were maintained on ice until analyzed

Positive controls for the PI, DHE, annexin binding, and caspase assays were generated by heating yeast to 40 ^∘^C for three minutes and by exposing yeast to 120 mM acetic acid for five minutes. Negative controls were generated using liquid yeast cultures in log phase growth.

Flow cytometry of PI-stained cells revealed three peaks - high uptake, low uptake, and negative uptake. Cell death was identified as high PI uptake (Lin et al. 2008). Cell apoptosis was identified as low uptake of PI and positive staining with annexin V, and expression of related caspases was identified with FITC-VAD-FMK (Qi et al. 2003. CaspACE™ FITC-VAD-FMK In Situ Marker (Promega Corporation, Madison WI) is a fluorescent analog of the pan caspase inhibitor Z-VAD-FMK (carbobenzoxy-valyl-alanyl-aspartyl-[O-methyl]-fluoromethylketone) (Qi et al. 2003). The fluorescein isothiocyanate (FITC) group has been substituted for the carbobenzoxy (Z) N-terminal blocking group to create a fluorescent apoptosis marker. This structure allows delivery of the inhibitor into the cell where it irreversibly binds to activated caspases.

Flow cytometry was performed in the Duke Human Vaccine Institute Research Flow Cytometry Shared Resource Facility under the direction of Dr. Gregory D. Sempowski (Durham, NC). Flow cytometric studies were carried on a Becton Dickinson (San Jose, CA) LSRII cell analyzer flow cytometer using Excitation/Emission of 510/580nm, 532/610nm, and 488/530 nm for DHE, PI, and GFP/ CaspACE™ FITC-VAD-FMK/annexin, respectively. The instrument was aligned using fluorescent beads. Yeast cell gating was established using a display of log side scatter fluorescence verses linear forward scatter, using glutaraldehyde stained pan fluorescent controls for confirmation in fluorescent channels. Unstained yeast samples were used to determine levels of background fluorescence Measurements were collected on at least 10,000 cells per sample.

### Statistics

**Fig. 2:**
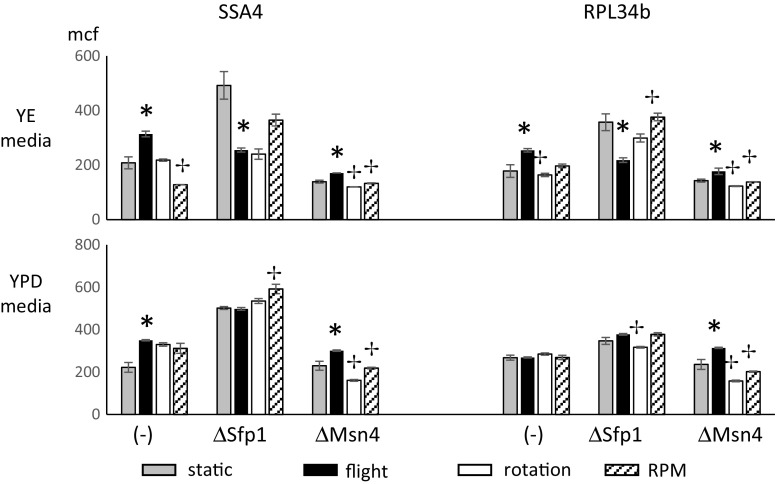
Role of Sfp1 and Msn4 in expression of SSA4 and RPL34B by giant yeast colonies grown in real and simulated microgravity. eGFP-expressing SSA4 (YER103W) or RPL34B (YIL052C) yeast clones, with and without deletion of the Sfp1 or Msn4 promoters, were inoculated onto ‘poor’ YE agar (upper panel) or YPD ‘rich’ agar (lower panel). Colonies were grown for 28 days under static (gray bars), flight (black bars), rotating wall vessel (rotation; white bars), or random positioning machine (RPM, hatched bars) conditions. Colonies were harvested, dispersed in saline, and analyzed by flow cytometry for eGFP intensity indicative of SSA4 or RPL34B expression. Data are the geometric mean of the mean channel fluorescence and error bars are the SEM of 4-12 replicates. Asterisks indicate clones where flight and static were significantly different; crosses indicate clones where rotation or RPM were significantly different from flight

**Fig. 3:**
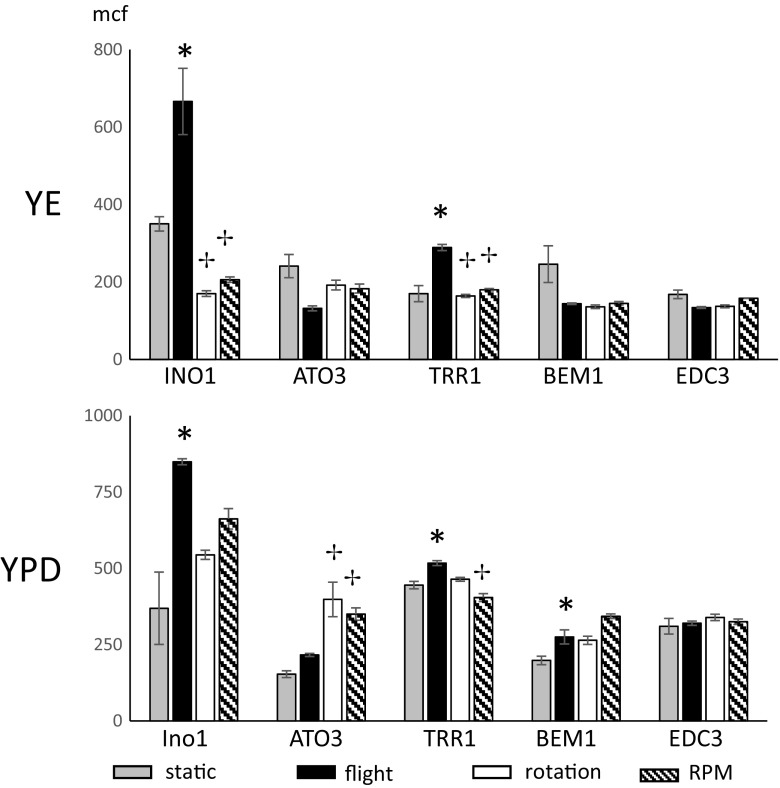
Expression of genes in giant yeast colonies grown in real and simulated microgravity. eGFP-expressing INO1, TRR1, BEM1, ATO3, EDC3 yeast clones, were inoculated onto ‘poor’ YE agar (upper panel) or YPD ‘rich’ agar (lower panel). Colonies were grown for 28 days under static (gray bars), flight (black bars), rotating wall vessel (rotation; white bars), or random positioning machine (RPM, hatched bars) conditions. Colonies were harvested, dispersed in saline, and analyzed by flow cytometry for eGFP intensity indicative of gene expression. Data are the geometric mean of the mean channel fluorescence and error bars are the SEM of 3-4 replicates for flight, rotation, and RPM and 7-8 replicates for static cultures. Asterisks indicate clones where flight and static were significantly different; crosses indicate clones where rotation or RPM were significantly different from flight

**Fig. 4:**
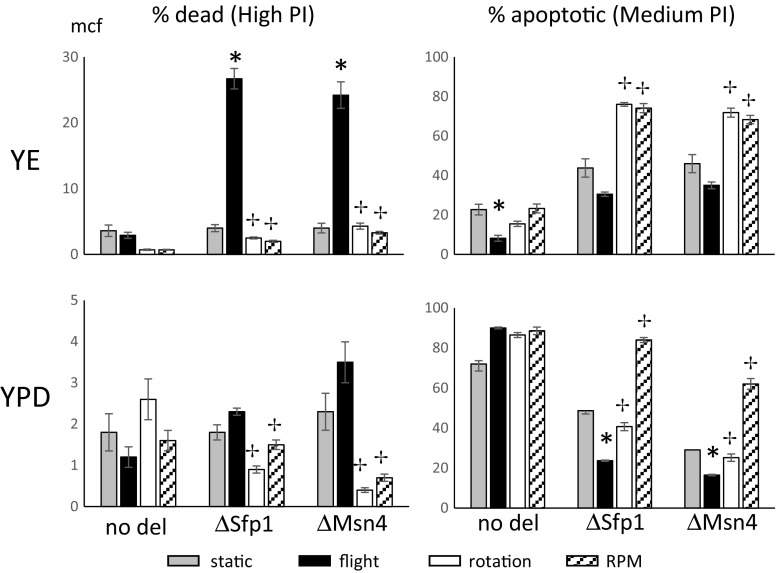
Role of Sfp1 and Msn4 in viability of giant yeast colonies grown in real and simulated microgravity. eGFP-expressing SSA4 (YER103W) or RPL34B (YIL052C) yeast clones, with and without deletion of the Sfp1 or Msn4 promoters, were inoculated onto ‘poor’ YE agar (upper panel) or YPD ‘rich’ agar (lower panel). Colonies were grown for 28 days under static (gray bars), flight (black bars), rotating wall vessel (rotation; white bars), or random positioning machine (RPM, hatched bars) conditions. Colonies were harvested, dispersed in saline and analyzed by flow cytometry for uptake of propidium iodide. Data are the % of yeast that were apoptotic (low propidium iodide uptake) or dead (high propidium iodide uptake) and error bars are the SEM of 8 to 24 replicates. Asterisks indicate clones where flight and static were significantly different; crosses indicate clones where rotation or RPM were significantly different from flight

Flow cytometry data is presented as geometric mean ±standard error of the mean of fluorescence in arbitrary units compared to a negative control with a minimum of six replicates. Geometric means were determined using FlowJo Software (Ashland, OR). Analysis of variance and post-hoc comparison using Tukey’s test was performed using Statistica 6.1 (StatSoft Inc., Tulsa, OK), using correlation matrix product moment and partial correlations. Flow cytometry data was also analyzed statistically using Kolgomorov-Smirnov summation statistics (Young 1977). Specific fluorescent values and statistical parameters for the data in Figs. 2, 3, and 4 are tabulated in Tables 2, 3, and 4.

**Table 2:** Role of Sfp1 and Msn4 in expression of SSA4 and RPL34B by giant yeast colonies grown in real and emulated microgravity

Clone	average	average	average	average	ANOVA	Tukey Static	Tukey Static	Tukey Static	Tukey Flight	Tukey Flight	Tukey RWV
	static	Flight	RWV	RPM		vs. Flight	vs. RWV	vs. RPM	vs. RWV	vs. RPM	vs. RPM
YE media											
Ssa4	208 ± 22	314 ± 11	218 ± 4	128 ± 1	0.0001	0.004	ns	0.03	ns	0.0002	0.04
Sfp1Δ Ssa4	492 ± 51	255 ± 7	240 ± 19	365 ± 22	0.0004	0.003	0.001	ns	ns	ns	ns
Msn4Δ Ssa4	139 ± 5	171 ± 0	120 ± 1	133 ± 2	0.0001	0.001	0.04	ns	0.0002	0.0009	0.33
YIL052c	178 ± 23	254 ± 7	164 ± 6	197 ± 7	0.04	0.05	ns	ns	0.04	ns	ns
Sfp1Δ RPL34B	357 ± 31	218 ± 9	299 ± 15	376 ± 14	0.002	0.004	ns	ns	ns	0.005	ns
Msn4Δ RPL34B	143 ± 5	177 ± 12	123v2	138v1	0.0002	0.004	ns	ns	0.0002	0.004	ns
YPD media											
Ssa4	222 ± 23	350 ± 3	330 ± 8	312 ± 24	0.002	0.003	0.01	0.04	ns	ns	ns
Sfp1Δ Ssa4	502 ± 7	498 ± 6	535 ± 11	592 ± 11	0.00002	ns	ns	0.0002	ns	0.0003	0.02
Msn4Δ Ssa4	230 ± 21	301 ± 3	161 ± 4	219 ± 4	0.0004	0.03	0.04	ns	0.003	0.03	ns
YIL052c	268 ± 12	269 ± 3	285 ± 5	26 ± 11	ns	ns	ns	ns	ns	ns	ns
Sfp1Δ RPL34B	347 ± 16	378 ± 4	317 ± 4	378 ± 8	0.03	ns	ns	ns	0.04	ns	0.05
Msn4 Δ RPL34B	236 ±23	314 ± 3	158 ± 4	202 ± 3	0.0002	0.03	0.03	ns	0.0003	0.006	ns

eGFP-expressing SSa4 (YER103W) or RPL34B (YIL052c) yeast clones, with and without deletion of the Sfp1 or Msn4 promoters, were inoculated onto nutrient poor YE agar or YPD nutrient rich agar. Colonies were harvested after 28 days and assayed for expression of GFP by flow cytometry. Data are the geomean of the mean channel fluorescence ±error bars are the SEM of 4-12 replicates. p values are shown for ANOVA, followed by pairwise Tukey analyzes. ns - not significant, p > 0.05

**Table 3:** Expression of genes in giant yeast colonies grown in real and simulated microgravity

Clone	average	average	average	average	ANOVA	Tukey Static	Tukey Static	Tukey Static	Tukey Flight	Tukey Flight	Tukey RWV
	static	flight	RWV	RPM		v. Flight	v. RWV	v. RPM	v. RWV	v. RPM	v. RPM
YE Media											
INO1	350 ± 19	666 ± 85	170 ± 8	206 ± 7	0.00001	0.0002	0.003	0.02	0.0002	0.0002	ns
TRR1	170 ± 21	289 ± 8	164 ± 4	180 ± 3	0.002	0.002	ns	ns	0.003	0.008	ns
SSA4	208 ± 22	314 ± 11	218 ± 3	128 ± 1	0.0001	0.004	0.97	0.03	ns	0.0002	0.04
BEM1	246 ± 47	144 ± 2	136 ± 5	145 ± 5	ns	ns	ns	ns	ns	ns	ns
RPL34B	178 ± 23	254 ± 7	164 ± 6	197 ± 7	0.04	0.05	ns	ns	0.04	ns	ns
ATO3	241 ± 6	132 ± 6	192 ± 13	183 ± 12	ns	ns	ns	ns	ns	ns	ns
EDC3	168 ± 11	134 ± 2	137 ± 4	158 ± 1	0.05	0.09	0.09	ns	ns	ns	ns
YPD Media											
INO1	369 ± 119	849 ± 10	544 ± 15	662 ± 34	0.03	0.02	ns	ns	ns	ns	0.85
TRR1	445 ± 12	517 ± 8	464 ± 7	404 ± 13	0.0005	0.006	ns	0.1	0.07	0.0005	0.03
SSA4	222 ± 23	350 ± 3	330 ± 8	312 ± 24	0.002	0.003	0.01	0.04	ns	ns	ns
BEM1	198 ± 14	275 ± 23	264 ± 14	343 ± 8	0.00005	0.01	0.03	0.0002	ns	0.06	0.02
RPL34B	268 ± 12	269 ± 3	285+ 5	269 ± 11	ns	ns	ns	ns	ns	ns	ns
ATO3	153 ± 11	216 ± 5	39 ± 57	350 ± 21	0.00004	ns	0.0002	0.0007	0.006	0.045	ns
EDC3	310 ± 26	320 ± 7	339 ± 11	325 ± 9	ns	ns	ns	ns	ns	ns	ns

eGFP-expressing INO1, TRR1, SSA4, RPL34b, BEM1, and ATO3 yeast clones, were inoculated onto nutrient poor YE agar (upper panel) or YPD nutrient rich agar (lower panel). Colonies were harvested after 28 days and assayed for expression of GFP by flow cytometry. Data are the geomean of the mean channel fluorescence and error bars are the SEM of 3-4 replicates for flight, rotation, and RPM and 7-8 replicates for static cultures. p values are shown for ANOVA, followed by pairwise Tukey analyzes. ns - not significant, p > 0.05

**Table 4:** Role of Sfp1 and Msn4 in viability of giant yeast colonies grown in real and simulated microgravity

clone	average static	average flight	average RWV	average RPM	ANOVA	Tukey Static vs. Flight	Tukey Static vs. RWV	Tukey Static vs. RPM	Tukey Flight vs. RWV	Tukey Flight vs. RPM	Tukey RWV vs. RPM
YE media - High PI uptake											
no del	3.6 ± 0.9	2.9 ± 0.5	0.7 ± 0.1	0.7 ± 0.1	0.01	ns	0.04	0.04	ns	ns	ns
Sfp Δ	4.0 ± 0.5	26.7 ± 1.6	2.5 ± 0.1	2.0 ± 0.1	0.00001	0.0001	ns	ns	0.0001	0.0001	ns
Msn4 Δ	4.0 ± 0.7	24.2 ± 2.0	4.3 ± 0.5	3.3 ± 0.2	0.00001	0.0002	ns	ns	0.0002	0.0002	ns
YE media - Low PI Uptake											
no del	23 ± 3	8 ± 2	16 ± 1	23 ± 2	0.0004	0.0001	ns	ns	ns	0.002	ns
Sfp1 Δ	44 ± 5	31 ± 1	76 ± 1	74 ± 2	0.00001	ns	0.0002	0.0002	0.0002	0.0002	ns
Msn4 Δ	46 ± 5	35 ± 2	72 ± 2	68 ± 2	0.00001	ns	0.0002	0.0009	0.0002	0.0002	ns
YE media - Apoptosis											
DHE	1286 ± 299	2056 ± 100	621 ± 55	680 ± 38	0.02	ns	ns	ns	ns	ns	
caspase	594 ± 17	668 ± 35	617 ± 11	787 ± 11	0.00003	ns	ns	0.0002	ns	0.01	0.0006
annexin	141 ± 8	221 ± 12	158 ± 5	164 ± 4	0.00004	0.0002	ns	ns	0.001	0.003	ns
YPD media - High PI uptake											
no del	1.8 ± 0.5	1.2 ± 0.2	2.6 ± 0.5	1.6 ± 0.2	ns	ns	ns	ns	ns	ns	ns
Sfp1 Δ	1.8 ± 0.2	2.3 ± 0.1	0.9 ± 0.1	1.5 ± 0.1	0.00007	ns	0.0005	0.477	0.0002	0.02	0.07
Msn4 Δ	2.3 ± 0.4	3.5 ± 0.5	0.4 ± 0.1	0.7 ± 0.1	0.00002	ns	0.003	0.01	0.0002	0.0002	ns
YPD media - Low PI Uptake											
no del	72 ± 8	90 ± 1	87 ± 1.	89 ± 2	ns	ns	ns	ns	ns	ns	ns
Sfp1 Δ	49 ± 4	24 ± 1	41 ± 2	84 ± 1	0.00001	0.0001	ns	0.0001	0.003	0.0001	0.0001
Msn4 Δ	30 ± 2	17 ± 1	25 ± 2	62 ± 3	0.00001	0.0002	ns	0.0002	0.02	0.0001	0.0001
YPD media - Apoptosis											
DHE	3109 ± 570	8778 ± 406	1398 ± 42	1459 ± 51	0.00001	0.0002	ns	ns	0.0002	0.0002	ns
caspase	1047 ± 59	1403 ± 74	1059 ± 39	1133 ± 46	0.01	0.008	ns	ns	0.02	ns	ns
annexin	389 ± 31	373 ± 5	525 ± 17	493 ± 14	0.006	ns	0	ns	0.03	ns	ns

SSA4 (YER103W) or RPL34B (YIL052c) yeast clones, with and without deletion of the Sfp1 or Msn4 promoters, were inoculated onto ‘nutrient poor’ YE agar or ‘nutrient rich’ agar YPD. Colonies were harvested after 28 days, dispersed in saline and analyzed by flow cytometry for uptake of PI. Data from SSA4 and RPL34B clones with no promoter deletion were pooled, data from SSA4 and RPL34B with Sfp1 deletions were pooled, and data from SSA4 and RPL34B with Msn4 deletions were pooled. PI uptake data are the % of yeast that were apoptotic (%low PI uptake) or dead (% high PI uptake) and error bars are the SEM of 8-24 replicates. SSA4 and RPL43B clones without promoter deletions were also assayed for ROS with DHE and for apoptosis by expression of caspase and binding of annexin. Data are the geomean channel fluorescence and error bars are the SEM of 8 replicates. p values are shown for ANOVA, followed by pairwise Tukey analyzes. ns - not significant, p > 0.05

## Results

Expression of the stress response genes, SSA4 and RPL34b, was measured using eGFP fusion protein expression clones. Stress promoter dependence was assessed by comparing the eGFP signal of the parent strains to clones in which the Msn4 or Sfp1 promoter genes had been deleted (Fig. 2 and Table 2).

In summary, in colonies grown on rich YPD media, SSA4 increased compared to static culture in all culture modalities. The increases in SSA4 expression seen in flight and rotation, but not random positioning, were Sfp1 dependent. RPL34B gene expression did not change during any culture condition on YPD. Flight and rotation had oppositely directed RPL34B gene expression changes dependent on Msn4.

Colonies grown on nutrient poor YE media yielded gene expression changes distinct from those seen in nutrient rich YPD media. SSA4 expression increased in flight and was Sfp1dependent. Ssa4 expression did not change during rotation and decreased during random positioning. In both rotation and random positioning, expression of SSA4 was dependent on both Msn4 and Sfp1. On YE media, RPL34B increased in flight with Msn4 dependence, but did not change during rotation or random positioning.

Expression of ATO3, INO1, TRR1, BEM1 and EDC3 is shown in Fig. 3 and Table 3. Expression of SSA4 and RPL34B is shown in Fig. 2, as noted above. Expression of the EDC3 gene did not change expression during any culture modality, demonstrating that we are not observing a field change. Expression of three genes, INO1, TRR1, and SSA4, changed in flight on both YPD and YE. In flight, expression of RPL34B increased on YE but not YPD, while expression of BEM1 was the reverse, changing in increasing in YPD but not YE. The only other change in INO1 was a decreased expression on YE during rotation. Expression of TRR1 had no other changes. Expression of SSA4 also decreased during random positioning on YE, and increased on YPD during rotation and random positioning. Expression of BEM1 did not change on YE, but increased on YPD with flight, rotation and random positioning. ATO3 expression, which did not change in flight, had no changes on YE, but increased on YPD under both rotation and random positioning growth conditions.

Cell death by necrosis and apoptosis (as measured by PI and Annexin V staining) was affected by the culture modalities and manifested diverse stress defense promoter dependence (Fig. 4 and Table 4). In summary, in flight cell necrosis had no baseline changes but showed dramatic Sfp1 and Msn4 dependence only on YE. Necrosis was reduced on YE during both rotation and random positioning dependent on both Sfp1 and Msn4. Basal necrosis rates were decreased and dependence on Sfp1 during rotation, and on Msn4 during both rotation and random positioning. In flight, apoptosis was reduced only on YE, and on YPD basal apoptosis was both Sfp1 and Msn4 dependent. During rotation basal apoptosis rates were both Sfp1 and Msn4 dependent only on YE. During random positioning basal apoptosis rates were both Sfp1 and Msn4 dependent on both YE and YPD.

Changes in Annexin V exposure and caspase activity (indicative of apoptosis) were consistent with the changes in the middle peak of propidium iodide stained cells (e.g. low PI uptake) observed by flow cytometry. The specific fluorescent values and statistical parameters for DHE staining are tabulated in Table 4. As controls, assay of fresh wild type cells was no different to unstained cells for annexin binding but showed caspase expression providing a negative control baseline (data not shown). Treatment with acetic acid or heat induced both caspase activity and annexin binding providing positive controls (data not shown). Annexin only changed from static controls during rotation on YE, and on YPD only in flight. Stress defense promoter dependence was not tested. Caspase only changed from static controls during flight on YE, and on YPD only during rotation.

Reactive oxygen species (ROS) production, especially superoxide formation, was monitored with DHE. Positive control cells were induced with acetic acid and heat (data not shown). On YE, none of the culture techniques changed DHE signal, but basal levels were Sfp1 and Msn4 dependent. The specific fluorescent values and statistical parameters for DHE staining are tabulated in Table 4. On YPD, DHE signal was increased in flight, independent of the stress defense promoters. Rotation and random positioning did not change DHE levels, but basal levels were both Sfp1 and Msn4 dependent under these culture conditions.

## Discussion

Systematic studies of the effects of true microgravity and common microgravity simulations on cell physiology has been lacking. Furthermore, how these environments combine with other fundamental variables such as growth media to influence the cellular response to stress is an outstanding question. We address both of these by examining cellular changes, including gene expression (reported by shear stress promoters Msn4 and Sfp1), reactive oxygen species, and apoptosis.

The current studies define a role of ammonia signaling and stress pathways using physical methods to disrupt ammonia convection in giant yeast colonies. Several lines of evidence support increased ammonia signaling during the near total lack of convection during colony culture in microgravity. First, of all the models tested, microgravity was the only one to increase both select reactive oxygen species and stress defense genes. These genes include inositol-3-phosphate synthase (INO1), thioredoxin reductase (TRR1), and stress seventy subfamily A (SSA4). These changes were independent of media type. Interestingly, both INO1 and TRR1 are predominantly expressed in the L or lower colony cells when yeast colony stratify into feeder and growing layers in giant colonies (Cap et al. 2009a; Palkova et al. 1997). This suggests that ammonia signaling is increased when the generated ammonia cannot convect away from the colonies. Second, the increased expression of the stress defense dependent SSA4 gene is consistent with lack of oxygen availability and carbon dioxide accumulation as these gasses are also affected by the lack of convection. Third, expression of both SSA4 and RPL34B increased more in flight when the classic stress defense promoter Msn4 was deleted. Shear stress promoters have a complex interplay of effects on the expression of genes they modify (Coleman et al. 2007, 2008a, b; Hammond et al. 1999; Johanson et al. 2002). Table 5 summarizes the effects of shear press promoters on expression of dependent genes during manipulation of physical forces from this report as well as previous studies. Even within just these two shear stress response genes (SSA4 and RPL34b), with and without deletion of shear stress promoters (Msn4 and Sfp1), there is scant commonality of the gene expression changes or promoter dependence. Interestingly, a similar signaling phenomenon was recently reported for bacteria, where acetate accumulation in the boundary layer affected metabolism, presumably likewise due to lack of convection in microgravity (Zea et al. 2016) and in *E. coli* where nutrient depletion in simulated microgravity induced stress response genes (Vukanti and Leff 2012).

**Table 5:** Dependence of SSA4 and RPL34B on Msn4 and Sfp1 under different conditions

Condition	Msn4 dependence		Sfp1 dependence		Study
	SSA4	RPL34B	SSA4	RPL34B	
1g Static - Rich media, liquid	No	No	No	Yes	Coleman et al. (2008a)
Magnetic levitation - Rich media, liquid	Yes	Yes	Yes	Yes	Coleman et al. (2007)
Neutral buoyancy - Rich media, liquid	Yes	No	Yes	No	Coleman et al. (2008b)
Spaceflight - Rich media, liquid	Yes	Yes	No	No	Coleman et al. (2008a)
RWV - Rich media, liquid	Yes	Yes	Yes	No	Coleman et al. (2008b)
1g Static - Rich media, plated	No	No	Yes	Yes	this report
RPM - Rich media, plated	Yes	Yes	Yes	Yes	this report
RWV - Rich media, plated	Yes	Yes	Yes	No	this report
Spaceflight - Rich media, plated	No	Yes	Yes	Yes	this report
1g Static - Poor media, plated	Yes	No	Yes	Yes	this report
RPM - Poor media, plated	No	Yes	Yes	Yes	this report
RWV - Poor media, plated	Yes	Yes	No	Yes	this report
Spaceflight - Poor media, plated	Yes	Yes	Yes	Yes	this report

Cell death by necrosis and apoptosis increased in flight as shown by multiple modalities: uptake of propidium iodide up, expression of caspase, and binding of annexin V. Induction of apoptosis by simulated microgravity has been seen with other cell types including human lymphocytes (Ward et al. 2006). This increased cell death was greatly increased by deletion of either of the stress responsive promoters Msn4 or Sfp1. The finding of both necrotic and apoptotic cell death is further evidence for activity of both ammonia signaling and stress defense pathways, as ammonia signaling utilizes mostly caspase independent pathways (Vachova and Palkova 2005) and stress defense is mediated mostly by apoptosis as well as autophagy and necrosis (Farrugia and Balzan 2012).

The changes in INO1 and SSA4 genes are significant in both random positioning with gravity vector averaging and rotation techniques but oppositely directed to the space flight changes, underscoring the uniqueness of the effects of true microgravity. During culture on the ISS, yeast cultures are also exposed to increased radiation (Nislow et al. 2015). Our previous studies using yeast deletion series in liquid culture to evaluate the effects of spaceflight revealed two dominant responses: radiation/DNA repair and reactive oxygen pathways (Nislow et al. 2015). We conclude that the ISS provides a unique suite of stimuli with distinct set of responses: the question is how to translate these properties to i) further NASA’s program objectives, ii) support commercial development, and iii) address unmet clinical needs.

We found differences in the responses of yeast in the rotating wall vessel versus random positioning models in both ammonia-dependent and stress-dependent changes, providing clarity to the controversy as to whether the random positioning device is a form of rotation, or a distinct stimulus (Klaus 2001; Klaus et al. 2004). These differences included gene expression changes mediated by shear stress promoters, redox status, and apoptosis. Hence, we conclude that the forces induced by random positioning and rotation are distinct. Many resources are available for scientists seeking to study the effects of gravity on biologic systems (Brungs et al. 2016; Frett et al. 2016) and researchers should be mindful that fluidity of the culture milieu and shear stress at the membrane surface are likely to be important variables affecting how cells sense gravity (Kohn et al. 2017; van Loon 2008). In our model system composed of a near-solid colony of yeast, receiving nutrients from an agar gel at its base, and using a gas, ammonia, as one means of driving differentiation within the colony, spaceflight, rotating wall vessel and random positioning generate different effects. In free-floating liquid systems such as U937 cells growing in suspension (Bradamante et al. 2006), HUVEC cells adherent to Cytodex beads (Bradamante et al. 2006), or thyroid cells growing as spheroids (Warnke et al. 2016) rotating wall vessel, random positioning machines, and spaceflight had very similar effects.

The ability of physical methods to manipulate yeast colony redox, apoptosis, and stress defense status is important at multiple levels of organismal biology. Gene deletion, although targeted to a single locus, will often cause follow-on effects in multiple integrated pathways, especially when promoters are deleted (Park et al. 2011). Further, there are clinically important applications. Analysis of yeast survival pathways offers powerful techniques to address drug metabolic mechanisms for repurposing of pharmaceuticals (Lee et al. 2014), including identification of unique targets (Blackman et al. 2012) and buffering pathways required to respond to drugs. Further, yeast colony differentiation recapitulates many of the stratification mechanisms of tumorigenesis (Cap et al. 2012a, d; Birdsall et al. 2016). In conclusion, by allowing graded manipulation of a combination of convection and ammonia signaling, while avoiding secondary effects of gene deletion, these physical techniques are primed to substantially contribute to our understanding of mechanisms of drug action, cell aging, and colony differentiation.

## Abbreviations

2-D: 2 dimensional
3-D: 3 dimensional
ATO3: Ammonia (Ammonium) Transport Outward 3 (YDR384C)
BEM1: Bud Emergence (YBR200W)
BY4743: Yeast strain with genotype MATa/α his3Δ1/his3Δ1 leu2Δ0/leu2Δ0 LYS2/lys2Δ0 met15Δ0/MET15 ura3Δ0/ura3Δ0
CaspACE™ FITC-VAD-FMK: carbobenzoxy-valyl-alanyl-aspartyl-[O-methyl]-fluoromethylketone
DHE: Dihydroethidium
EDC3: Enhancer of mRNA DeCapping (YEL015W)
Em: Emission
Ex: Excitation
g: gravity
GFP: green fluorescent protein
INO1: inositol-3-phosphate synthase 1(YJL153C)
ISS: International Space Station
Msn4: Multicopy suppressor of SNF1 mutation 4 (YKL062W)
ORF: Open reading frame
PBS: Phosphate buffered saline
PHAB: Plate habitat
PI: Propidium iodide
ROS: Reactive oxygen species
RPL34B: Ribosomal Protein of the Large subunit (YIL052C)
RPM: Random positioning machine
SEM: Standard error of the mean
Sfp1: Split Finger Protein 1 (YLR403W)
Ssa4: Stress-Seventy subfamily A4 (YER103W)
TRR1: thioredoxin reductase 1 (YDR353W)
WT: Wild type
YE: Yeast extract (“nutrient poor media”)
YPD: Yeast peptone dextrose (“nutrient rich media”)
